# 环境污染物的跨胎盘转移效率研究进展

**DOI:** 10.3724/SP.J.1123.2024.07002

**Published:** 2025-01-08

**Authors:** Keyu YUAN, Jun XIONG, Bifeng YUAN

**Affiliations:** 武汉大学公共卫生学院, 湖北 武汉 430071; School of Public Health, Wuhan University, Wuhan 430071, China

**Keywords:** 色谱-质谱, 环境污染物, 跨胎盘转移效率, 全氟和多氟烷基物质, 多溴二苯醚, 多氯联苯, 有机氯农药, 综述, chromatography-mass spectrometry, environmental pollutants, transplacental transfer efficiency (TTE), per- and polyfluoroalkyl substances (PFASs), polybrominated diphenyl ethers (PBDEs), polychlorinated biphenyls (PCBs), organochlorine pesticides (OCPs), review

## Abstract

随着工业化发展,环境污染物的种类和数量不断增加。环境污染物广泛存在于空气、食品和生活用品等传播介质中,妊娠期接触环境污染物不仅影响孕妇健康,还可能对分娩结果及胎儿的发育产生不利影响。胎盘作为母体和胎儿之间的屏障,对部分环境污染物具有选择性阻隔作用,即一些污染物被阻留于母体血液,而另一些环境污染物通过被动扩散、胎盘转运蛋白或内吞作用穿过胎盘屏障,进入胎儿脐带血中。跨胎盘转移效率(transplacental transfer efficiency, TTE)即污染物在脐带血中与在母体血液中水平的比值,常用于评估环境污染物跨越胎盘屏障的能力。TTE与污染物的相对分子质量、脂溶性、极性和结构特征等理化性质有关;母体和胎儿的健康状况、胎盘血流量、胎盘成熟度、胎盘转运蛋白和代谢酶的功能也会显著影响污染物的转移过程。本文总结了TTE的研究方法以及近年来有关环境污染物(全氟和多氟烷基物质(PFASs)、多溴二苯醚(PBDEs)、多氯联苯(PCBs)和有机氯农药(OCPs)等)的TTE研究进展,并讨论了影响TTE的主要因素。本文对环境污染物的跨胎盘转移机制研究具有指导意义,并有助于评估环境污染物对孕妇和胎儿健康风险的影响。

## Abstract (en)

Industrialization has led to significant increases in the types and quantities of pollutants, with environmental pollutants widely present in various media, including the air, food, and everyday items. These pollutants can enter the human body via multiple pathways, including ingestion through food and absorption through the skin; this intrusion can disrupt the production, release, and circulation of hormones in the body, resulting in a range of illnesses that affect the reproductive, endocrine, and nervous systems. Consequently, these pollutants pose substantial risks to human health. In particular, fetuses are highly sensitive to environmental pollutants during critical stages of development, and exposure during periods of growth and development can result in more-obvious and severe health hazards that can lead to preterm birth, low birth weight, and fetal malformations. The placenta acts as a barrier between the mother and fetus, and selectively filters certain pollutants. While some pollutants remain in the maternal bloodstream, others cross the placental barrier into the fetal umbilical blood through passive diffusion, placental transport proteins, or endocytosis. The transplacental transfer efficiency (TTE) is the ratio of the level of the pollutant in the umbilical blood to that in the maternal blood, and is a valuable metric for evaluating the ability of a pollutant to breach the placental barrier. A higher TTE implies that a larger proportion of pollutants are transferred from the mother to the fetus, thereby amplifying the potential risks to the fetus. Mass spectrometry-based detection methods are extensively used in the chemical and environmental sciences because they are exceptionally sensitive and highly resolving. This analytical technique involves ionizing compounds within a sample and identifying them based on their distinct mass-to-charge ratios; it enables both qualitative and quantitative analyses of various environmental pollutants. Current methodologies for examining the TTE of a pollutant include in-vitro experiments, animal studies, epidemiologic studies, and model calculation; these approaches help to evaluate the transfer of pollutants from mother to fetus via the placenta. Analyzing the TTEs of different chemicals enables high-risk pollutants to be identified and provides an understanding of their abilities to cross the placenta. Research on the transplacental transfer of environmental pollutants has focused mainly on per- and polyfluoroalkyl substances (PFASs), polybrominated diphenyl ethers (PBDEs), polychlorinated biphenyls (PCBs), and organochlorine pesticides (OCPs), with relatively few studies on the TTEs of other pollutants reported. Pollutant transfer through the placenta is a complex process that is influenced by factors that include the physical and chemical properties of the pollutant (e.g., molecular mass, solubility, and lipophilicity), maternal factors (e.g., maternal health and lifestyle, maternal genetics, environmental conditions, and socioeconomic status), and placental characteristics (e.g., placental maturity, placental blood flow, transport proteins, and metabolic enzymes). This review summarizes recent advances in research on the TTEs of environmental pollutants, focusing on analytical methods, the TTEs of PFASs, PBDEs, PCBs, and OCPs, and the pivotal factors that influence TTEs. Studying the TTEs of pollutants enables their characteristics to be elucidated, thereby providing support data for research on the exposure, transfer, and accumulation of pollutants in the human body, as well as a theoretical framework for understanding the mechanism of transplacental transfer of environmental pollutants. This research is expected to play a vital role in assessing the impact of environmental pollutants on the health of pregnant women and their fetuses.

随着农业生产技术和现代工业技术的不断发展,环境污染物已经渗透到我们的生产、生活中,并广泛分布于空气、食品和生活用品中^[1⇓-3]^。环境污染物可以通过呼吸道、消化道和皮肤暴露等途径进入人体,并随血液循环进入各个组织脏器中。部分污染物及其代谢产物能够在人体内蓄积,干扰人体的正常生理功能,从而导致人体生殖系统、内分泌系统和神经系统产生各种疾病。例如,全氟和多氟烷基物质(PFASs)会干扰人体内分泌系统,引起机体代谢紊乱;多溴二苯醚(PBDEs)会对甲状腺激素的分泌水平产生影响;多氯联苯(PCBs)和有机氯农药(OCPs)会对儿童神经系统发育产生不利影响。由此可见,污染物的长期、低剂量暴露会对人体健康造成重大威胁^[4⇓⇓-7]^。

妊娠期是指女性从怀孕开始到分娩结束的整个过程,一般持续40周左右。在妊娠期间,孕妇的生理状态会发生剧烈变化,包括激素水平、血液循环系统和免疫系统等,这些变化都会影响环境污染物在孕妇体内的暴露水平,并增加孕妇对环境污染物的敏感度,使孕妇在妊娠期间更容易受到环境污染物的不良影响^[8]^。妊娠期接触有毒化学物质不仅会危害孕妇健康,增加糖尿病等患病风险^[9]^,还会影响胎儿发育和分娩结果,造成不良妊娠结局,如早产、低出生体重、畸形等^[10⇓-12]^。胎盘由胎儿部分的羊膜和叶状绒毛膜以及母体部分的底蜕膜构成,是维持胎儿生长发育的重要器官,具有物质交换、防御保护、代谢及免疫功能等多重关键作用^[13⇓-15]^。由于胎盘的存在,部分污染物会被阻留于母体血液中,而部分污染物则能够穿过胎盘屏障进入胎儿血液,导致胎儿在子宫内直接接触环境污染物^[16,17]^。跨胎盘转移效率(transplacental transfer efficiency, TTE)是污染物在脐带血中与在母体血液中水平的比值,可用于评估污染物穿透胎盘屏障的能力。TTE的数值越高,表示污染物从母体转移到胎儿体内的比例就越多,进而对胎儿健康的影响就越大^[18,19]^。本文总结了TTE的研究方法以及近年来有关环境污染物(PFASs、PBDEs、PCBs和OCPs等)的TTE研究进展,并讨论了影响TTE的主要因素。研究污染物的TTE有助于揭示污染物的性质,为污染物在人体内的暴露、转移及蓄积研究提供数据支持,并为理解环境污染物的跨胎盘转移机制奠定理论基础,这对于评估环境污染物对孕妇及胎儿的健康影响具有重要指导意义。

## 1 环境污染物的色谱-质谱检测方法

在计算TTE之前,首先要对人体中环境污染物的水平进行高灵敏度检测和准确定量。基于色谱-质谱的检测技术具有分离能力强、灵敏度高、定量准确和稳定性好等优势,已广泛应用于环境污染物的检测^[20]^。质谱通过将样品中的化合物离子化,并根据质荷比的差异来分析和识别这些化合物,可用于各种环境污染物的定性和定量分析。常用的色谱-质谱检测技术主要有气相色谱-质谱(GC-MS)和液相色谱-质谱(LC-MS)^[21⇓⇓⇓⇓⇓-27]^。

### 1.1 GC-MS

GC-MS是一种高效的分析方法,常用于环境污染物的检测,它结合了气相色谱的高效分离能力和质谱的高灵敏度与高特异性,可对各种有机污染物进行快速、准确的定性和定量分析^[28⇓-30]^。例如,Kumar等^[31]^利用GC-MS测定了孕妇血液样本中的多环芳烃,分析了母体多环芳烃暴露与新生儿低出生体重之间的关系;Foster等^[32]^使用GC-MS测定了孕妇血液样本中的PBDEs,分析了母体PBDEs暴露与新生儿出生体重之间的关系。GC-MS适用于挥发性、半挥发性和持久性有机污染物的分析检测,然而对于极性较大的环境污染物,GC-MS的分析能力较弱。此外,GC-MS的样品前处理过程较为复杂且耗时较长,在预处理、分离和检测过程中可能会出现误差,从而影响最终定量结果。

### 1.2 LC-MS

LC-MS常用于血清、血浆和尿液中有机污染物以及其他各种化合物的分析检测^[33⇓⇓⇓⇓⇓⇓⇓-41]^。例如,Uldbjerg等^[42]^使用LC-MS测定了孕妇尿液样本中的两种环境污染物(酚类化合物和邻苯二甲酸酯代谢物),并分析了孕期环境污染物暴露与新生儿出生结局之间的关系。Ma等^[43]^使用LC-MS测定了孕妇血浆中的PFASs,分析了亲本血浆中污染物暴露与体外受精结果之间的关系。LC-MS适用于极性、中性和疏水性有机污染物的分析检测,能够对目标化合物进行准确定性和定量分析;LC-MS的分析速度较快,可同时分析多种化合物,提高样品分析效率,但其质谱图比对过程可能会影响鉴定的准确性。本课题组^[44]^针对目前环境污染物TTE的研究现状,开发了一种基于液相色谱-串联质谱(LC-MS/MS)的污染物分析方法。该方法的样品前处理过程简便,生物样本用量少且灵敏度高,可对人体血清中的91种污染物进行同时定性和定量分析,其中包括13种双酚类化合物、25种阻燃剂、34种邻苯二甲酸酯和19种PFASs。

## 2 环境污染物的TTE研究方法

环境污染物的TTE研究涉及多种方法和技术,包括体外实验、动物实验、流行病学研究和模型计算等(图1),这些方法和技术可用于探究环境污染物如何从母体穿过胎盘转移到胎儿,以及评估环境污染物的转移程度。

**图1:**
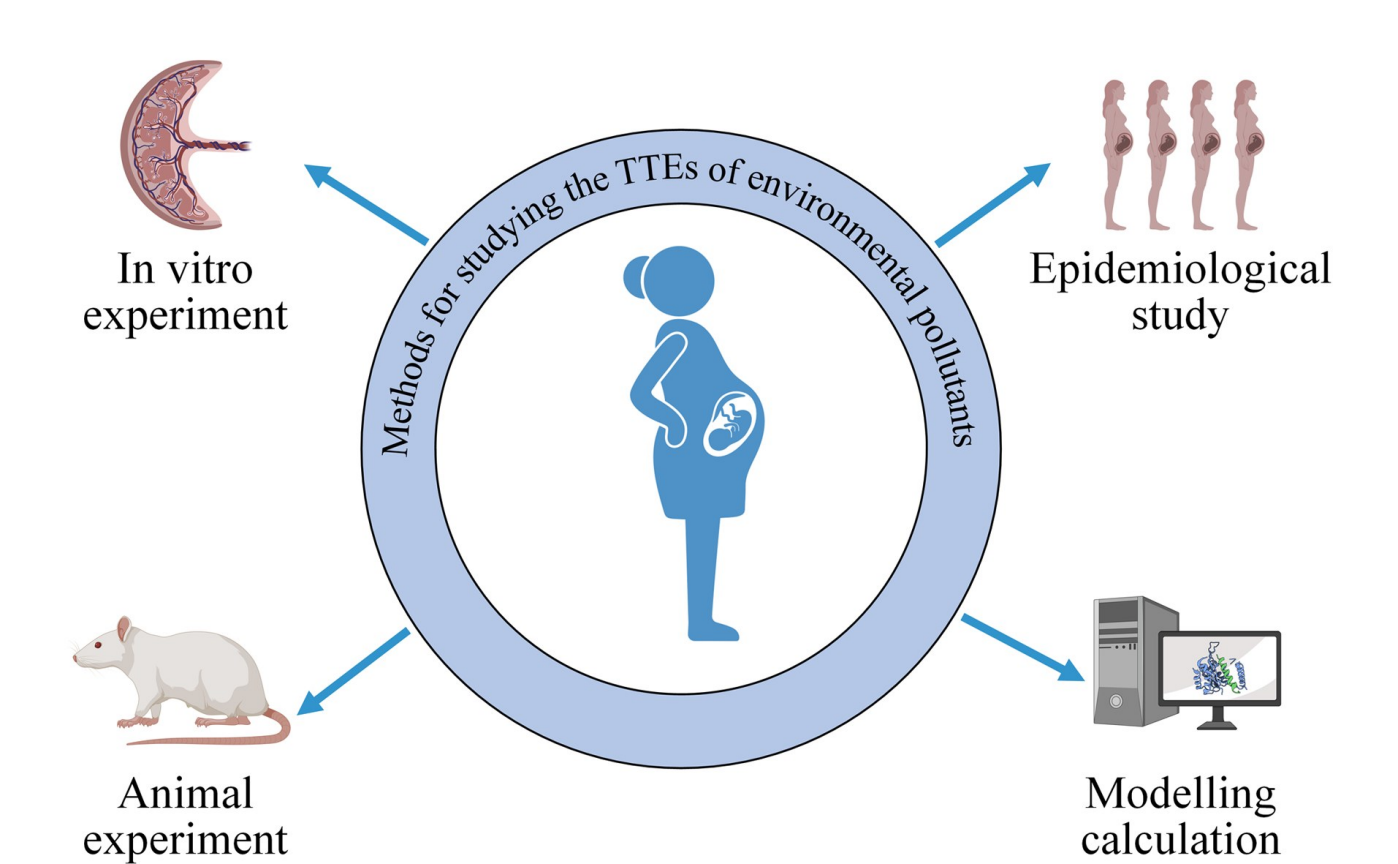
环境污染物的TTE研究方法

### 2.1 体外实验

胎盘灌流模型(placental perfusion model)是一种常用的污染物TTE体外实验方法^[45]^。该方法使用自然分娩或剖宫产获得的健康、完整且足月的人类胎盘,在体外模拟子宫内的环境条件,通过分析胎盘两侧灌流液的污染物水平来研究污染物的TTE。Frederiksen等^[46]^使用胎盘灌流模型对污染物的TTE进行了研究,该模型先通过产妇(一般是通过剖腹产手术)获取胎盘,随后进行体外灌流实验,利用母体和胎儿两侧的灌流液来模拟血液流动,监测污染物在母体与胎儿间的转移过程。胎盘灌流模型通过模拟胎盘的生理环境,提供了一种相对直接和可控的污染物跨胎盘转移研究方法,但该模型无法完全模拟体内的复杂环境,如生理和免疫反应等;并且,与体内实验相比,胎盘在体外状态下的新鲜度较低且会迅速退化,导致其功能和结构与体内状态不同,从而影响实验结果的准确性和可靠性。

### 2.2 动物实验

对于体内实验,常利用妊娠动物来研究污染物的TTE^[47]^。研究中常用的动物模型包括大鼠、小鼠和兔等,这些动物的生理和生殖特征与人类相似。动物实验中常采用灌胃染毒、注射染毒或经呼吸道染毒等方式构建污染物暴露模型,通过对母体血液、胎盘和胎儿组织中的污染物水平进行检测来研究污染物的TTE。Cai等^[48]^使用十溴二苯醚对大鼠模型进行暴露处理,探索了十溴二苯醚在母体和胎儿之间的转移过程,并在母体血液、胎盘和新生大鼠血液中均检测到了十溴二苯醚的代谢物。动物实验能够模拟复杂的人体环境,可以观察到污染物长期暴露对母体和胎儿的影响,包括生长发育、器官功能和行为变化等;然而,动物模型不能完全反映人类的胎盘特性,因此实验结果无法直接应用于人类,同时在进行此类实验时还需考虑动物伦理问题。

### 2.3 流行病学研究

除了上述两种方法,流行病学方法也可用于污染物的TTE研究^[12,49]^。流行病学方法可直接通过对人群的研究来获得污染物的TTE,通过问卷调查和环境监测等方式收集数据,对生物样本(如母体血液、脐带血和胎盘组织)进行检测,并计算污染物的TTE,同时还可以对污染物的暴露水平与TTE之间的相关性进行分析,能够较为全面地了解污染物暴露对母婴健康的影响。Li等^[18]^利用出生队列进行人群研究,对母婴血清中的PFASs进行了检测。实验结果发现,与传统的PFASs相比,PFASs替代品具有更高的TTE。该方法直接对人类受试者进行研究,所获得的实验结果更具有实际意义,但在研究过程中难以控制混杂因素且污染物在个体间的暴露水平差异较大,因此对样本量的要求较高。

### 2.4 模型计算

Li等^[50]^使用生理学基础药代动力学模型和分子动力学模拟来研究污染物在胎盘细胞膜中的穿透和转运机制。生理学基础药代动力学模型使用数学方程来描述污染物在体内的吸收、分布、代谢和排泄特征,并结合污染物的半衰期等数据来调整参数;通过建立母体-胎盘-胎儿系统模型,模拟污染物的分布和转移,研究污染物的TTE。Guan等^[51]^构建了以胎盘基因网络为中心的新型计算模型,该模型能够探究胎盘内多种转运体的功能,并评估化合物穿过胎盘屏障的能力;通过机器学习算法,该模型还能够计算出化合物穿过胎盘屏障的概率分数,从而进行系统评估。然而,该方法仅能提供可能的数据和研究方向,需要在复杂的体内环境中进一步验证。

## 3 主要环境污染物的TTE

环境污染物的TTE研究主要集中于几种已知的环境污染物,包括PFASs、PBDEs、PCBs和OCPs等。例如,本课题组^[44]^利用LC-MS/MS技术,研究了母婴人群中双酚类化合物、阻燃剂、邻苯二甲酸酯和PFASs的TTE,并分析了胎盘转运体与污染物TTE之间的关系。

### 3.1 PFASs

PFASs是指分子结构中至少含有一个全氟化甲基或亚甲基碳原子的一类化合物。PFASs具有独特的理化性质(如疏水性),已广泛应用于食品包装、灭火泡沫和家用产品等多个领域^[52,53]^。目前,在饮用水、化妆品等样品中均已检测到多种PFASs^[54]^,其中全氟辛基磺酸(PFOS)和全氟辛酸(PFOA)因具有持久性、生物富集性、危害性和长距离迁移性,已被列入《斯德哥尔摩公约》的持久性有机污染物清单中。人体监测实验也发现,在血液(包括全血、血清和血浆)、脐带血和母乳等样品中均存在PFASs^[55,56]^。研究证实,PFASs能够穿过胎盘屏障,经脐带血到达胎儿体内,此阶段的外源性污染物暴露可能对新生儿后期发育产生不良影响^[2]^。近年来,已有多项研究分析了PFASs的TTE,其中包括全氟丁酸(PFBA)、全氟己酸(PFHxA)、全氟庚酸(PFHpA)、PFOA、全氟壬酸(PFNA)、全氟癸酸(PFDA)、全氟十一酸(PFUnDA)、全氟十二酸(PFDoDA)、全氟十三酸(PFTrDA)、全氟十四酸(PFTeDA)、全氟丁基磺酸(PFBS)、全氟己基磺酸(PFHxS)和PFOS等^[43,56]^。据文献[57⇓⇓⇓⇓⇓-63]报道,上述PFASs的TTE平均值分别为2.17、2.71、3.40、0.82、0.59、0.35、0.36、0.76、1.68、1.90、0.97、0.62、0.39(见表1)。研究还发现,在配对的母体血清和脐带血清样本中,PFASs的水平还存在显著相关性(*P*<0.05)^[10]^。此外,PFASs的TTE与烷基链长、官能团种类、支链结点的位置以及对映体特征有关^[63]^。研究结果显示,PFASs的TTE随碳链长度的增加而减少,并呈现出“U”型趋势^[18]^。研究还发现,与具有相同碳链长度的全氟磺酸盐相比,全氟羧酸盐的TTE更大,因此更容易穿过胎盘屏障。此外,PFOS、PFHxS和PFOA的分支异构体相较于其线性异构体具有更高的TTE^[19]^。

**表1:** 主要环境污染物的TTE

Compound	Abbr.	TTEs						Refs.
		Mean	Minimum	P_25_	Median	P_75_	Maximum	
PFASs								
Perfluorobutanoic acid	PFBA	2.17	-	-	-	-	-	[60,62]
Perfluorohexanoic acid	PFHxA	2.71	-	-	-	-	-	[60,61]
Perfluoroheptanoic acid	PFHpA	3.40	-	-	-	-	-	[61,62]
Perfluorooctanoic acid	PFOA	0.82	-	-	-	-	-	[57,58]
Perfluorononanoic acid	PFNA	0.59	-	-	-	-	-	[57,61]
Perfluorodecanoic acid	PFDA	0.35	-	-	-	-	-	[57,61]
Perfluoroundecanoic acid	PFUnDA	0.36	-	-	-	-	-	[57,60]
Perfluorododecanoic acid	PFDoDA	0.76	-	-	-	-	-	[60,61]
Perfluorotridecanoic acid	PFTrDA	1.68	-	-	-	-	-	[58,59]
Perfluorotetradecanoic acid	PFTeDA	1.90	-	-	-	-	-	[61,62]
Perfluorobutanesulfonic acid	PFBS	0.97	-	-	-	-	-	[61,62]
Perfluorohexanesulfonic acid	PFHxS	0.62	-	-	-	-	-	[57,59,63]
Perfluorooctanesulfonic acid	PFOS	0.39	-	-	-	-	-	[57,59]
PBDEs								
2,4,4'-Tribromodiphenyl ether	BDE-28	-	-	0.52	0.77	1.18	-	[64,65]
2,2',4,4'-Tetrabromodiphenyl ether	BDE-47	-	-	0.60	0.84	1.51	-	[66]
2,2',4,4',5-Pentabromodiphenyl ether	BDE-99	-	-	0.44	0.62	1.00	-	[64,66]
2,2',4,4',6-Tribromodiphenyl ether	BDE-100	-	-	0.43	0.63	0.81	-	[64,66]
2,2',4,4',5,5'-Hexabromodiphenyl ether	BDE-153	-	-	0.29	0.41	0.54	-	[64,66]
2,2',4,4',5,6'-Hexabromodiphenyl ether	BDE-154	-	-	0.34	0.45	0.65	-	[66]
2,2',3,4,4',5',6-Heptabromodiphenyl ether	BDE-183	-	-	0.25	0.40	0.62	-	[67]
Decabromodiphenyl ether	BDE-209	-	-	0.25	0.55	0.89	-	[65,67]
PCBs								
2,3',4,4',5-Pentachlorobiphenyl	PCB118	-	0.44	-	-	-	1.94	[68⇓⇓⇓-71]
2,2',3,4,4',5'-Hexachlorobiphenyl	PCB138	-	0.42	-	-	-	1.45	[68⇓⇓⇓-71]
2,2',4,4',5,5'-Hexachlorobiphenyl	PCB153	-	0.71	-	-	-	1.25	[68,69,72,73]
2,2',3,4,4',5,5'-Heptachlorobiphenyl	PCB180	-	0.49	-	-	-	1.87	[68,72,73]
OCPs								
Hexachlorocyclohexane	HCH	-	-	-	1.09	-	-	[69]
Dichlorodiphenyltrichloroethane	DDT	-	-	-	1.11	-	-	[69,74,75]
Chlordane	CHL	-	-	-	0.65	-	-	[69]
Hexachlorobenzene	HCB	-	-	-	1.58	-	-	[69]

P_25_: 25th percentile; P_75_: 75th percentile; PFASs: per- and polyfluoroalkyl substances; PBDEs: polybrominated diphenyl ethers; PCBs: polychlorinated biphenyls; OCPs: organochlorine pesticides; -: unfilled.

### 3.2 PBDEs

PBDEs是一种由苯环和溴原子组成的有机卤代化合物。由于具有高阻燃性,PBDEs被广泛应用于电子产品、塑料、建筑材料以及家具等各类消费品中。然而,PBDEs具有生物富集性、生物毒性和潜在的内分泌干扰问题,已被《斯德哥尔摩公约》列为需逐步淘汰的化学品;此外,由于其具有环境持久性,人类长期处于PBDEs的暴露之中。多项研究^[65]^表明,2,4,4'-三溴联苯醚(BDE-28)、2,2',4,4'-四溴联苯醚(BDE-47)、2,2',4,4',5-五溴联苯醚(BDE-99)、2,2',4,4',6-五溴联苯醚(BDE-100)、2,2',4,4',5,5'-六溴联苯醚(BDE-153)、2,2',4,4',5,6'-六溴联苯醚(BDE-154)、2,2',3,4,4',5',6-七溴联苯醚(BDE-183)和十溴联苯醚(BDE-209)广泛存在于母体血液、脐带血和胎盘样本中,且不同PBDEs的TTE存在差异。据文献[64⇓⇓-67]报道,上述PBDEs的TTE中位数和四分位数间距分别为BDE-28(0.77, 0.66)、BDE-47(0.84, 0.91)、BDE-99(0.62, 0.56)、BDE-100(0.63, 0.38)、BDE-153(0.41, 0.25)、BDE-154(0.45, 0.31)、BDE-183(0.40, 0.37)、BDE-209(0.55, 0.64)(见表1)。研究表明,PBDEs在人体内可通过去溴或氧化反应降解,将高溴代同系物转化为低溴代化合物,然而这些PBDEs代谢产物的毒性可能比PBDEs更高;此外,与亲水性PBDEs相比,亲脂性PBDEs更容易穿过胎盘屏障^[65]^。

### 3.3 PCBs

PCBs是一类由苯环与氯原子连接而成的有机氯化合物,广泛用于电气设备、润滑油、密封剂、油漆和塑料制品等工业产品中。PCBs具有毒性和环境持久性,能够在环境和生物体中长期残留和富集。鉴于PCBs对环境和人类健康造成的影响,包括我国在内的许多国家和地区已采取相关措施,以限制或禁止PCBs的生产和使用。PCBs及其羟基化代谢物在人群样本中普遍存在。据文献[68⇓⇓⇓⇓-73]报道,多种PCBs能够穿过胎盘到达胎儿体内,包括2,3',4,4',5-五氯联苯(PCB118)、2,2',3,4,4',5'-六氯联苯(PCB138)、2,2',4,4',5,5'-六氯联苯(PCB153)和2,2',3,4,4',5,5'-七氯联苯(PCB180),它们的TTE分别为0.44~1.94、0.42~1.45、0.71~1.25和0.49~1.87(表1)。此外有研究^[65]^表明,相对分子质量较低的PCBs更容易从母体侧转移到胎儿侧,而相对分子质量较高的PCBs更倾向于在胎盘组织中蓄积。

### 3.4 OCPs

OCPs是一类高效且持久的杀虫农药,在20世纪中期得到了广泛应用。OCPs主要包括滴滴涕(DDT)、六氯环己烷(HCH)、六氯苯(HCB)、艾氏剂(ALD)、狄氏剂(DLD)和氯丹(CHL)。由于具有环境持久性、生物蓄积性以及广泛的环境和生物危害,许多OCPs已被大多数国家禁用或限制使用。尽管如此,DDT、HCH和HCB等OCPs仍能够在环境和人体中检出。据文献[69,74,75]报道,HCH、DDT、CHL和HCB的TTE中位数分别为1.09、1.11、0.65和1.58(表1)。此外研究结果显示,DDT、HCB和HCH在母体血清和脐带血(或胎盘)中的水平具有相关性^[75]^。

### 3.5 其他污染物

前文总结了4类环境污染物的TTE,这些是在相关研究中较为常见的4类污染物。相比之下,其他环境污染物(如阻燃剂、重金属等)的TTE研究较少。其他污染物的研究主要集中在母体或婴儿体内的污染物暴露水平评估,而对于污染物的TTE研究则关注较少。Kim等^[76]^对母婴中四溴双酚A(TBBPA)和六溴十二烷(HBCD)的暴露水平进行了检测,分析了污染物暴露与甲状腺功能减退之间的联系,但该研究未对两种污染物的TTE进行探索。因此,未来还需进一步加强对其他种类环境污染物的TTE研究。

## 4 TTE的影响因素

环境污染物的TTE是一个受多因素影响的复杂过程,其中污染物的理化性质(如相对分子质量、溶解度和脂溶性)以及母体的健康状况和生活方式都是影响TTE的关键因素。胎盘作为胎儿与外界环境之间的媒介,其生理特性(如转运蛋白和代谢酶)同样会对污染物的TTE产生显著影响。此外,母体的遗传背景以及环境和社会经济因素也可能会间接影响污染物的TTE。因此,深入研究这些因素对TTE的作用,对于预防和减少污染物对胎儿健康的潜在影响至关重要。

### 4.1 污染物的理化性质

环境污染物的理化性质是影响TTE的关键因素,包括相对分子质量、脂溶性、极性、电荷以及结构特征等。研究发现,相对分子质量小的污染物更容易穿过胎盘屏障,它们能够通过简单扩散或胎盘上的小孔从母体侧转移至胎儿侧^[60,77]^。与低脂溶性污染物相比,高脂溶性污染物更容易穿过胎盘屏障,因为胎盘屏障含有脂质层,这些脂质层对脂溶性化合物有较高的溶解度,其中部分脂溶性污染物还会在胎盘组织内蓄积。此外,与极性污染物相比,非极性和中等极性污染物的TTE通常更高;极性污染物和带电污染物在跨胎盘转移过程中更容易受到胎盘电荷屏障的阻挡,或需要依赖特定的转运蛋白来协助其转移^[68,78]^。污染物的分子结构(如环状结构或长链结构)也可能会影响其与胎盘细胞膜之间的相互作用,进而影响其TTE^[10,76]^。例如,随着碳链长度的增加,PFAS的TTE会呈现出“U”型趋势;具有羧酸结构的PFAS更容易通过胎盘屏障;与线性异构体相比,分支异构体具有更高的TTE等。

### 4.2 母体和胎儿因素

污染物的TTE在很大程度上受到母体和胎儿的生理特性和健康状况的影响。母体血液中的污染物水平直接决定了污染物跨胎盘转移的驱动力,高暴露水平往往导致更高的转移效率;同时,不同地区的环境污染物暴露水平差别较大,因此TTE也会产生差异。母体的健康状况(如肝肾功能、营养状态等)会影响污染物在母体内的分布和代谢,从而影响其TTE^[19,60,79]^。此外,胎儿的健康状况也会影响污染物在胎儿侧的蓄积情况,早产和足月同样会影响污染物在母婴之间的分布^[10,18,60]^。

### 4.3 胎盘因素

胎盘的结构与功能完整性对于污染物的TTE起着决定性作用,胎盘的血流量、成熟度以及胎盘转运蛋白和代谢酶的功能等因素都会影响污染物在母体与胎儿之间的转移过程。高血流量会促进污染物在母体和胎儿之间的交换,胎盘屏障越薄,其通透性就越高,污染物也就越容易通过;此外,随着妊娠的进展,胎盘的结构和通透性也会发生变化,进而会影响污染物的转移过程。胎盘中存在两种主要的转运体(图2),一种是ATP结合转运体(ATP-binding cassette subfamily, ABC),包括P-糖蛋白(P-gp)、乳腺癌耐药蛋白(BCRP)和多药耐药相关蛋白(MRP);另一种是溶质载体家族(solute carrier organic anion transporter family, SLC),其中包括有机阴离子转运多肽(OATP)、有机阴离子转运体(OAT)和有机阳离子转运体(OCT)。这些转运体会对外源性化合物的跨胎盘转移过程产生显著影响,而转移过程又受到单个或多个不同转运体之间相互作用的复杂调控;此外,这些转运体也可以主动运输某些污染物,从而影响污染物在母体和胎儿之间的分布^[10,80,81]^。胎盘中存在多种代谢酶,如细胞色素P-450酶家族、谷胱甘肽转移酶等,它们能够将污染物进行代谢转化,使其更容易或更难穿过胎盘屏障^[48,60,66]^。某些污染物在母体中经过初级代谢后,可能会生成具有不同TTE的代谢产物,其中水溶性代谢产物更容易被排出,而非脂溶性代谢产物可能更容易穿过胎盘。此外,胎盘自身也具备一定的代谢能力,可以对某些污染物进行次级代谢,从而改变污染物的TTE及其在胎盘中的水平。

**图2:**
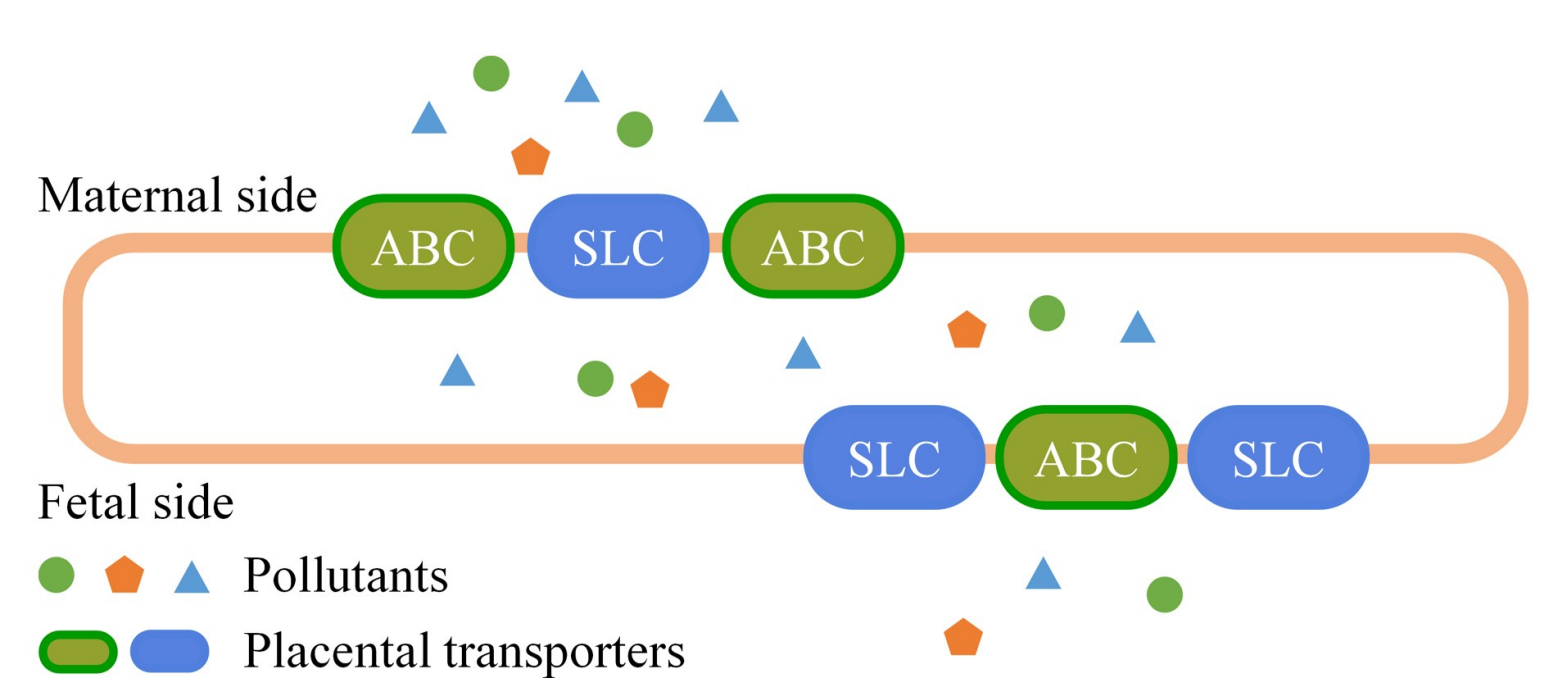
胎盘转运体转移环境污染物的示意图

## 5 总结与展望

环境污染物的TTE与胎儿的健康状况密切相关,暴露于环境污染物可能会导致胎儿发育异常和免疫系统紊乱,甚至增加出生缺陷的风险。胎儿在发育的关键阶段对环境污染物非常敏感,因此对多种化学物质的TTE进行研究,有助于明确污染物的转移能力,进而制定减少孕妇暴露的策略,降低污染物对胎儿的健康危害。

目前,TTE的研究主要集中在几种已知的环境污染物上,特别是PFASs。然而,人群还会接触到其他多种化学物质,但关于这些物质的TTE和胎儿暴露情况的研究却很少。尽管单一环境污染物的暴露水平较低,但人群处于多种污染物的联合暴露状态下,其中许多污染物之间具有协同作用,它们会通过干扰转运蛋白作用、抑制胎盘发育或诱导胎盘发生氧化应激和凋亡来损害胎盘的功能,并改变其对有毒化学物质的渗透性。因此,需要全面开展对多种环境污染物TTE的研究,以揭示胎盘的屏障功能和选择性转运机制。研究显示,环境污染物在胎盘中的蓄积与代谢过程可能会影响它们向胎儿侧的转移;由于胎盘组织中的脂质含量超过10%,使得亲脂性化学物质容易在胎盘内蓄积;此外,胎盘中含有多种酶,能够代谢多种物质。因此,在研究环境污染物的TTE时,应综合考虑胎盘的蓄积能力和代谢能力。此外,对胎盘转运蛋白和代谢酶的作用进行研究,有助于揭示胎盘对污染物的选择性转运机制,这对于理解环境污染物对胎儿健康和出生结果的影响至关重要。

未来,应当强化TTE的基础研究,发展高灵敏的污染物检测技术以提高污染物的检测能力,并通过多学科交叉合作来深入理解污染物的化学特性和胎盘的生理功能。例如,表观遗传修饰能够影响包括胎盘转运体在内的蛋白质表达水平,借助各种高灵敏、高精度的表观遗传修饰分析方法^[82⇓⇓⇓⇓⇓⇓⇓⇓⇓⇓⇓⇓⇓⇓⇓-98]^,研究人员能够探索环境污染物如何通过改变表观遗传修饰来影响胎盘转运体的表达。同时,应开展大规模的人群流行病学研究,结合风险评估模型,预测污染物对胎儿健康的影响,从而更好地评估和管理环境污染物的跨胎盘转移风险,为环境健康领域的研究与实践提供坚实的基础和明确的指导方向。
